# Dr. Trenor and Dental Colleges

**Published:** 1852-04

**Authors:** 


*Dr. Trenor and Dental Colleges.*—Our readers may have expected from us some review of Dr. Trenor's essay upon dental education, and his philippic against dental colleges. But we feel no disposition to give more attention to it than a bare notice. If Dr. Trenor's article proves any thing, it demonstrates the necessity of dental colleges, both argumentatively in what he says, and illustratively in the manner in which he says it. If the colleges can be overturned by such an onset as his, they must be feeble structures, and if the professors could be annoyed by his detraction they must be more sensitive than wise. Men sometimes in their ungovernable animosity, strike so hard as to blunt their own weapons, and benumb their own hands.

We would be sorry to do any thing to prevent Dr. Trenor's promotion. We think it will do him good and no body else any harm. He will never understand himself nor his scheme until he has tried to carry his views into practice. Disappointment may reach, where warning cannot, and mortification accomplish what argument has labored in vain to perform. For the present, Prejudice, the deaf man, sits at ear gate. Vanity seems to have been tickled into hysterics, and judgment granted a holy-day. After a while things will be better. "Anger is a ***brief*** madness," and self-conceit soon exhausts its swim bladder.

